# A Qualitative Exploration of the Mental Health and Psychosocial Contexts of HIV-Positive Adolescents in Tanzania

**DOI:** 10.1371/journal.pone.0165936

**Published:** 2016-11-16

**Authors:** Megan K. Ramaiya, Kristen A. Sullivan, Karen O' Donnell, Coleen K. Cunningham, Aisa M. Shayo, Blandina T. Mmbaga, Dorothy E. Dow

**Affiliations:** 1 Center for Health Policy & Inequalities Research, Duke Global Health Institute, Duke University, Durham, North Carolina, United States of America; 2 Department of Psychology, University of Washington, Seattle, Washington, United States of America; 3 Department of Social Medicine, University of North Carolina at Chapel Hill, Chapel Hill, North Carolina, United States of America; 4 Center for Child & Family Health, Duke University, Durham, North Carolina, United States of America; 5 Department of Pediatrics, Division of Pediatric Infectious Diseases, Duke University Medical Center, Durham, North Carolina, United States of America; 6 Kilimanjaro Christian Medical Centre, Moshi, Tanzania; The University of New South Wales, Neuroscience Research Australia, AUSTRALIA

## Abstract

Although 85% of HIV-positive adolescents reside in sub-Saharan Africa, little is known about the psychosocial and mental health factors affecting their daily well-being. Identifying these contextual variables is key to development of culturally appropriate and effective interventions for this understudied and high-risk population. The purpose of this study was to identify salient psychosocial and mental health challenges confronted by HIV-positive youth in a resource-poor Tanzanian setting. A total of 24 qualitative interviews were conducted with a convenience sample of adolescents aged 12–24 receiving outpatient HIV care at a medical center in Moshi, Tanzania. All interviews were audio-recorded, transcribed, and coded using thematic analysis. Psychosocial challenges identified included loss of one or more parents, chronic domestic abuse, financial stressors restricting access to medical care and education, and high levels of internalized and community stigma among peers and other social contacts. Over half of youth (56%) reported difficulties coming to terms with their HIV diagnosis and espoused related feelings of self-blame. These findings highlight the urgent need to develop culturally proficient programs aimed at helping adolescents cope with these manifold challenges. Results from this study guided the development of *Sauti ya Vijana (The Voice of Youth)*, a 10-session group mental health intervention designed to address the psychosocial and mental health needs of HIV-positive Tanzanian youth.

## Introduction

In 2012, there were 2.1 million adolescents living with HIV, with nearly 85% living in sub-Saharan Africa (SSA) [1]. The number of HIV-positive adolescents continues to grow due to increased survival of perinatally infected youth with access to antiretroviral treatment (ART) and high HIV incidence rates among this age group [2]. The period of adolescence is marked by significant biological, psychological, and psychosocial transitions, rendered more difficult by the addition of an HIV diagnosis [3]. Compared to younger children and adults, HIV-positive adolescents demonstrate consistently higher rates of poor adherence to ART and virologic failure[4–6]. Adolescents living with HIV must navigate a host of psychosocial difficulties that include considerable internalized and community stigma. Stigma, in turn, leads to added challenges negotiating sexual and personal identities common during this important developmental period [7, 8]. In SSA, chronic poverty and orphanhood are common experiences among HIV-positive adolescents [9–11], who are often tasked with adult responsibilities, including caregiving for family members, often resulting in irregular school attendance [12].

Given this range of psychosocial challenges, HIV-positive adolescents are particularly vulnerable to mental health problems [13–16] that have been shown to exacerbate poor ART adherence, leading to increased HIV-related morbidity and mortality [10]. In one United States sample, an estimated 44% of HIV-positive youth 16–21 years of age suffered from chronic depression one year following HIV diagnosis [17]. Another study in Uganda found that approximately 50% of HIV-positive adolescents reported significant psychological distress [13], and 18% of the participants reported a suicide attempt within the last year. HIV-positive adolescents are at heightened risk for post-traumatic stress following a range of potentially traumatic events including acute or prolonged domestic violence, sexual abuse, caregiver abandonment, and receiving a diagnosis of HIV [15, 16]. In one study by Radcliffe and colleagues [18], HIV-positive youth endorsed an average of 5.63 lifetime potentially traumatic events, a value that was increased relative to community samples. For 47% of surveyed youth, trauma-related symptoms were attributable to traumatic events beyond reception of an HIV diagnosis.

Despite the numerous psychosocial and mental health difficulties confronted by HIV-positive adolescents, there is a dearth of literature exploring the mental health burden encountered by these youth in SSA, with notable exceptions from South Africa focused on children orphaned by HIV/AIDS (both HIV-positive and negative) [9–11, 19–25] and adolescents in Rwanda [26] and Zambia [27]. To our knowledge, only one prior study in Zimbabwe has explored the psychosocial context of HIV-positive adolescents experiencing mental health problems [28]. This mixed-methods study is the only published study that investigated psychological or psychosocial well-being in HIV-positive youth in Tanzania [14].

Few evidence-based mental health treatments have been implemented and evaluated with HIV positive adolescents in SSA. Although rollout of the World Health Organization's Mental Health Gap Action Programme (mhGAP), an initiative aimed at eliminating the mental health treatment gap in low- and middle- income countries (LMIC) is currently underway [29], a substantial gap between mental health services need and availability remains. In some LMIC, this gap exceeds 90% [30]. In Tanzania, an official mental health policy is designed to integrate mental health into primary care [31]; however, few centers receive in-service training on mental health, and trained mental health professionals are scarce. In Moshi, Tanzania, Kilimanjaro Region where this study took place, there are currently no clinical psychologists or psychiatrists. Use of trained lay counselors to facilitate a trauma-focused cognitive-behavioral therapy with fidelity has been successful among orphans with grief symptoms in Tanzania [32]. A similarly manualized and scalable intervention for HIV-positive adolescents that can be integrated into the HIV adolescent clinic is urgently needed.

The present study was designed to identify and explore common psychosocial problems among HIV-positive adolescents in Tanzania who self-reported depressive and/or trauma-related symptoms, with the intent of using these qualitative data to inform development of a targeted mental health intervention for this population.

## Materials and Methods

### Study Design and Measures

This study was part of a larger mixed-methods project examining mental health difficulties among adolescents and young adults living with HIV in Tanzania [14]. Inclusion criteria for youth were: 1) ages 12 to 24 years; 2) receiving HIV medical care at Kilimanjaro Christian Medical Centre (KCMC) in Moshi, Tanzania; and 3) awareness of their HIV positive status. Youth living in institutions or orphanages were excluded from participation, as were those with significant cognitive impairment or developmental disability that could preclude understanding of informed consent/assent and study questions.

Depression (PHQ-9) [33] and posttraumatic stress (PTSD-RI) [34] symptoms were evaluated as part of a larger structured questionnaire. These measures were chosen because the PHQ-9 is a screener that has been frequently used with adult populations in SSA [35–38] and is recommended for use with adolescent populations [39] using a previously established cutoff score of 10 or greater as consistent with symptoms of depression [36, 37]. Posttraumatic stress symptoms were assessed with the University of California Los Angeles (UCLA) PTSD Exposure Screener and Reaction Index (PTSD-RI) [34, 40]. The PTSD-RI has been demonstrated to have good reliability and validity among youth in Zambia [41] and Kenya [42]. A modified four-point scale was utilized (range 0–51) here, with a positive symptom threshold of ≥ 18 [41].

A convenience sample of 62 HIV-positive youth who had enrolled in the larger quantitative study *(n = 182*) participated in a semi-structured interview designed to assess the psychosocial contexts of participants’ lives. Of those interviewed, participants who scored ≥10 on the PHQ-9 or ≥ 18 on the PTSD-RI were included in these analyses reflecting the likelihood of a significant number of self-reported mental health difficulties.

The semi-structured interview guide was informed by a review of the literature on psychosocial issues affecting HIV positive youth in SSA, consultation with medical and mental health providers serving this population in the United States and in the Kilimanjaro region, and by the biopsychosocial model [43]. Based on these sources, questions were developed to explore the following areas with adolescents: living situation and household dynamics, orphan status and prior or current relationships with parents, emotional wellbeing and coping strategies, attitudes towards HIV, attitudes towards ART and adherence, social support, daily stressors, HIV disclosure, intimate relationships, experiences with violence, and perspectives on the hospital-based adolescent HIV clinic called Teen Club. The guide was translated into the local language (Swahili) by two research assistants (both native Tanzanians) and back-translated by a native Swahili speaker who is bilingual in English to ensure accurate translation and verify any discrepancies. The Swahili interview guide was then reviewed with local interviewers and two youth-based focus groups and adapted for clarity.

### Data Collection

Four trained research assistants who spoke fluent Swahili conducted the in-depth interviews. In-depth interviews were usually conducted by the same research assistant who conducted the original structured survey. Youth were interviewed individually in a private room at KCMC to ensure confidentiality. Interviews were digitally recorded, and all participating youth were aware and consented to being recorded before the interview began.

### Data Analysis

Interviews were transcribed, translated, and reviewed for accuracy. QSR NVivo software version 10.0 was utilized for data management and analysis. The analytic approach was informed by thematic analysis [44]. All of the English transcripts were carefully reviewed by two members of the research team (MKR and KAS), with both identifying patterns to inform codebook development through this collaborative deep review, with multiple revisions as the understanding of the data was refined. When the codebook was finalized, 20% of the transcripts were double coded with discrepancies discussed until consensus between the researchers was reached. Themes emerged as comparisons within and across individuals were made, and representative quotations for each of the identified themes were selected.

### Ethics

Written informed consent was obtained prior to participation in the study by youth 18 years or older, or by a parent or guardian for youth less than 18 years of age. Youth under 18 years provided assent. All parts of the study were approved by the Institutional Review Boards of Duke University Medical School and Kilimanjaro Christian Medical University College as well as the National Institute of Medical Research in Tanzania.

## Results

### Participants

A total of 24 adolescent interviews were included in the present study, based on the established criteria for mental health difficulties. The average age of participants was 18 years, with a range of 13 to 23 years. Seventy-five percent of respondents were female. Of the 24 participants, 13 (54%) were currently enrolled in secondary or vocational school. The average number of years since HIV diagnosis for this sample was approximately 7 (range = 1–12).

### Living with HIV

#### Acceptance of HIV status

Many respondents discussed how they struggled to come to terms with finding out they were HIV-positive. Fifty four percent (*n = 13*) of adolescents reported feeling "different" from other youth because of their HIV status. Feelings of powerlessness about living with HIV were commonly expressed.

**P:** I don't feel like a normal human being. I feel like I am not a normal person because of this…sometimes I think about where I got it and wish God can take it away from me. Most of the time I get sick and think this disease is going to kill me. This disease is strong.

Another participant described sadness, self-blame, and feelings of isolation related to living with HIV.

**P:** During the past month, I was just feeling like I was weak, because I was in thoughts about…things, I stay alone, I cry myself. So I would feel weak, I feel lonely though I am surrounded by people. Sometimes I think…it's my fault because I have HIV.

However, 11 participants (46%) indicated they were able to come to terms with their status over time, in large part because they knew or associated with others who also had the disease:

**P**: In the beginning I felt so bad because I didn’t expect it. When I went to test I had confidence that I would be fine. I was so sad because I never expected or even thought about it. But now I feel fresh (normal), I am used to it and I meet different people who have the same problem, which makes me feel normal…

#### Spirituality

Six adolescents, all females (*n = 6*), made direct reference to a higher power when asked about sources of strength and support and/or how they feel about being HIV positive. One participant described her faith in God supporting her, despite ongoing economic hardship in her household:

**I:** Do you think that one day you will be able to continue with school?

**P:** Yes, when everything is settled and I got time I will go back to school and pay for myself because no one will pay for me.

**I:** Where will you get school fees?

**P:** I know God will provide

**I:** Do you have a job now?

**P:** No.

**I:** Who do you depend on now?

**P:** I depend on God, that’s why I live.

Others *(n = 2*), however, struggled to make sense of God’s plan with regard to their HIV status:

**I:**… do you have another thing that causes you stress?

**P:** At home where we live, we don’t have food most of the time and when my friends pass by and see how we live they laugh at us, I don’t really understand. I cry to God and say I wish we could have a life like others. I don’t know what God wanted that I was born with this disease.

#### Medication adherence and HIV education

In addition to the positive role of spirituality in some adolescents' lives, the availability of and adherence to ART was described as a protective factor. One third (*n = 8*) of adolescents cited the availability of life-prolonging ART as a positive means of coping with the psychosocial challenges described above. Similarly, support from HIV counselors and clinic staff in the form of HIV education was identified as a positive resource:

**I:** How do you feel about taking your medications?

**P:** I feel good because I know without these drugs I wouldn’t be here.

**I:** Why don’t you have any difficulties when it comes to take your drugs?

**P:** Because I know once I stop taking them I will have a lot of problems. I will feel so bad and sometimes when I forget to take them, I will feel so guilty that I haven’t done something important in my life that day.

### Domestic and Family Environments

#### Loss of parent(s)

Twenty-one percent of participants (*n = 5*) were double orphans, while 58% (*n = 14*) had only one living parent. Participants referenced multiple challenges resulting from the loss of one or more parents, including financial instability and maltreatment by current caregivers. Two participants also lamented the absence of a unique parent-child bond as an ongoing challenge:

**P:** As a young person, there are a lot of things that I think I haven’t gotten from my father. There are things that a father would have taught me, or given me advice as a father and son, but I don’t get them now. You know there are those things that only a parent can explain to the children and the children will understand them.

The majority of participants who had lost one or both parents described the situation as emotionally painful:

**P:** I lived mostly with my mother, but when my mother died I was devastated because she was everything to me. After she died I thought that was the end of my life. Her death affected me so much that I have not been able to get past that…In my mind, I cry all the time.

#### Experienced stigma

For some respondents, stigmatization by caregivers and/or other family members manifested in the separation of household utensils and similar items and by taunting and ridiculing about their diagnosis. Four participants reported unequal distribution of finances and emotional support compared to non-HIV-positive siblings or household members. A number of respondents attributed this discrimination to a lack of education regarding HIV transmission and progression.

**I:** Has it (*HIV*) affected your relationship with other people?

**P:** It has affected me because my aunts are avoiding me, when I call them they don’t pick up and they even say that now that I am sick it’s better that I stay with my mother so she can take care of me. I think that they are afraid that I can infect their children.

#### Domestic violence

A majority (*n = 18*) of respondents indicated that they were either currently experiencing violence in their household or had in the past. For a number of participants, violent acts included a combination of regular physical beatings and emotional abuse. Many adolescents cited difficulties living with family members who failed to acknowledge their needs and concerns and who often resorted to abusing them after using alcohol. All participants who had experienced current or past abuse identified these situations as distressing:

**P:** My uncle is the one who gives me stress a lot. I think a lot, and I get angry. My heart beats faster like someone has thrown water on me. It makes me cry a lot. Even if I hear his voice my heart becomes so sad it doesn’t matter if I was happy, but when I hear his voice my happiness disappears. When I hear my uncle is coming, then all the peace is gone.

**P:** When [my aunt] beats me I cry. After she is done beating me I lock myself in my room and cry a lot. I feel sad and frail in my heart.

#### Financial stress

Many adolescents (*n = 15*) indicated that ongoing financial stress was a significant burden in their lives. For a number of participants (*n = 7*), insufficient household funds and long-term financial uncertainty negatively affected their ability to attend medical appointments and collect their ART, leading to periodic gaps in medication adherence. For others, financial difficulties prevented them from either enrolling in or continuing in school. A number of participants struggled with this reality:

**P:** The first thing that brings me stress is school. I wish I can get someone who can take me back to school again. I will be so happy. I have been staying at home for a long time and I am worried that my mind is not growing at all. I really want to go to school.

### Social and Peer Environments

#### HIV stigma

Anticipated and experienced stigma at the peer and community level emerged as salient stressors to 71% (*n = 17*) of participants. These adolescents expressed concern and fear of ridicule, gossiping, and social isolation because of their HIV-positive status. A number of participants also reported experiencing acts of discrimination in both school and community settings. At school, stigma was largely perpetuated by classroom peers and occasionally by teachers. One participant noted experiencing HIV-related bullying in her usual circle of friends:

**P**: It has affected me so much. For example, sometimes when I quarrel one of my friends she will start saying that I have so many wounds on the face and that I will not live long enough. So it has affected my relationship with my good friends.

#### Disclosure difficulties

Stigma-related challenges also manifested during discussion of disclosure difficulties. Many (*n = 7*) adolescents reported going to great lengths to mask their HIV status amidst their peers; behaviors ranged from covert medication use, avoiding other identified HIV-positive peers, or sharing little regarding their family history.

**I:** Is there someone who knows that you are HIV positive?

**P:** No, other people are only guessing because I am taking drugs (ARVs).

**I:** Do they see you taking medication?

**P:** Sometimes we will be watching TV and I will send my young brother to go and bring me my drugs (ARVs). They ask if I am taking painkillers. I say yes, but I think they are guessing.

Only twenty-five percent of adolescents (*n = 6*) had disclosed their status to someone outside their immediate family, including friends, current, and potential romantic partners. Several participants (*n = 8*) expressed worries and reluctance about disclosure in the context of intimate relationships. Of those who had disclosed to partners, the outcomes were varied: two respondents had HIV-negative partners who reacted with acceptance, while the partners of two others either terminated the relationship or responded with extreme anger. In the following excerpt, one respondent describes her fear of entering a romantic relationship:

**I:** And how has that affected your relationship with other people?

**P:** With my relatives it hasn’t affected anything, but with other people it has. For example, I can’t be in any intimate relationship with anyone because I don’t know his status and I just can’t tell every guy who wants to be with me that I am HIV positive. I need time and courage to do that.

Another participant explained that her fear of disclosure has negatively affected her sense of self-worth:

**I:** How do you feel that you are HIV positive? How do you see yourself?

**P**: I feel terrible and I keep asking myself how can this be possible? I feel like I will never have my own child? Who will marry me? And if I will get a man, what if he will ask me to do the test and find out that he is negative and I am positive? What will happen if I will get married one day?…I have no confidence. That’s why I can’t trust new people in my life.

For a number of participants (*n = 9*), the fear of disclosure reinforced their sense of social isolation, leading to emotional distress and further self-isolating behaviors:

**P:** I feel so bad especially since I am left alone. I can't share this with anyone. I wish I had a friend or sibling from the same parents or even aunts and uncles, but I don’t have any. I am alone and it hurts me a lot.

#### Sexual and witnessed violence

Eight adolescents, all of whom were female, reported past incidences of sexual violence perpetrated by community members. Three participants reported non-consensual intercourse. Two adolescents also indicated that there were insufficient legal and social structures in place to protect individuals who had experienced sexual violence.

Thirty-eight percent (*n = 9*) of participants also reported having witnessed violence, with some describing anxiety and painful memories surrounding the events. One participant explains:

**I:** Have you witnessed somebody beaten?

**P:** Yes.

**I:** What happened?

**P:** Thieves were caught at my neighborhood. They were beaten to death right there.

**I:** Were you watching that?

**P:** Yes.

**I:** Did you know them?

**P:** Yes, I knew them. They are people whom I used to pass on the street but I never thought they were thieves…Whenever I think about it I feel chills in my body, the way they cut them with the machetes it was beyond crazy. I get shivering whenever I think about that.

#### Social support

In spite of the many challenges described above, peer support also emerged as instrumental in aiding positive coping among HIV-positive adolescents. Three adolescents who reported positive peer relationships reported less HIV-related distress and barriers, compared to respondents citing a specific lack of social support (*n = 8*). At the peer level, respondents citing one or more close social relationships (*n = 4*) indicated they felt open in sharing problems, seeking advice, and reaching out for other forms of help. In the following quote, one participant describes the close connections she formed in her school setting:

**I:** Do you have any other relationship that’s important to you?

**P:** I have found so many friends in college right now and I really treasure their friendship to me, I don’t want anything to happen to our friendship because it’s very hard to find real friends these days so I want to continue having them as my friends even after we finish our studies.

In addition to peer support, two adolescents also openly discussed the positive impact the hospital-based HIV clinic for adolescents, Teen Club, had on their sense of connectedness to others. In addition to receiving medical and clinical support, respondents spoke of the positive relationships they developed during the time in Teen Club:

**I:** Do you like coming to the teen clinic?

**P:** Yes, very much. Even when I am in college I really miss it because I miss my friends that I am so used to, and we help each other a lot by giving ourselves advice.

**I:** What kind of help do you get from other teens?

**P**: I get a lot of advice from them. Last clinic day we discussed a topic about disclosure, that when we have a boyfriend/girlfriend is it good if we can tell them the truth or we should wait until they find out themselves? Most of them said we shouldn’t tell them, but I said we should tell them what we have because if you love someone we have to be honest with them. But we have to see first if he/she also love you back because you can’t just tell anyone that said he/she love you. You have to monitor that person and make sure you know his behavior well enough so that he will not be able to tell anyone about your status… I like my friends, especially when we all meet together. It’s the only thing that makes me come here.

## Discussion

This study describes the reports of Tanzanian adolescents living with HIV with depressive and/or traumatic symptoms, used to understand mental health needs towards intervention development. Congruent with prior research in other SSA settings, youth expressed challenges with the loss of a parent(s), financial stressors limiting access to medical care and school, and high levels of stigma among peers and other community members [25, 45–48]. Some described self-blame and a lack of acceptance around their HIV positive status, while others, often citing social support and other Persons Living With HIV/AIDS (PLWHA) in their social networks, were able to accept their diagnosis.

Results of this study informed the development of a group mental health intervention, *Sauti ya Vijana (The Voice of Youth)*. The intervention model addresses the findings from the qualitative study about the stressors of youth living with HIV/AIDS to support them moving forward with fewer mental health symptoms, improved relationships, and better medication adherence. The model was designed to incorporate evidence-based practices of trauma-focused cognitive behavioral therapy [49], interpersonal psychotherapy [50], and motivational interviewing [51] into 10 group sessions with two joint caregiver sessions and two additional individual sessions. The intervention is designed to identify feelings, normalize and validate common stressors and worries, and to teach youth ways to relax and cope with stresses in life. The cognitive behavioral triangle is used for youth to understand and practice how thoughts influence emotions and subsequent behaviors. Principles of interpersonal psychotherapy used in the intervention are adapted for youth to identify the people in their support system as well as how they can remember and tell the story line about the potentially traumatic event of learning their HIV diagnosis. The memories are shared, first, with the lay counselor in an individual session (a strategy identified by [32]), second, with the youth group (as much as is desired by the youth), and third, with the caregiver. Approaches to motivation interviewing are applied to help youth reduce stigma, prepare for HIV disclosure to others, and to identify their values and plan for changes in behavior or thinking necessary to their motivations. The evidence-based principles are combined so that the intervention model, if successful in reducing symptoms and increasing ART adherence in the pilot randomized trial, can be scaled up in other low-resource settings with lay counselors.

Given that only adolescents with depressive and/or trauma-related symptoms were included in this analysis, we would anticipate finding more stressors and fewer sources of support than in a sample without these criteria. Nonetheless, the abuse suffered by the majority of respondents perpetrated by caregivers and/or other family members is significant. While prior research has documented maltreatment of orphans by caregivers to be common [52], youth orphaned by AIDS or living with HIV-infected caregivers are more likely to experience emotional and physical abuse than non-affected youth [53]. Results from this Tanzanian population are reflected and in prior research (e.g., [54, 55]) and are encouraging for the generalizability of the intervention developed to address these mental health needs.

This qualitative study has limitations. The respondents were a convenience sample of adolescents engaged in HIV care and therefore not necessarily representative of HIV-positive adolescents in the area. This sample included youth from 12 to 24 years old, representing a wide developmental span. Additionally, while gender differences were acknowledged when notable, explicit gender-based analyses were beyond the scope of this work. Future research with a larger sample should explore psychosocial trajectories among this population, potentially examining the influence of salient factors including age, gender, and orphan status, to provide insight into similarities and variation within and among these youth as they proceed into early adulthood. Such insight can inform tailored, developmentally appropriate interventions to promote mental and physical health and wellbeing.

These findings illustrate the complexity of the lives of HIV-positive adolescents in Tanzania and, likely, those in other SSA countries. Interventions to support mental health and HIV treatment adherence among this population facing complex psychosocial issues are urgently needed. These findings informed the development of an evidence-informed group-therapy intervention facilitated by lay counselors to address and treat symptoms of trauma, depression, and emotional and behavioral difficulties in a way that is scalable and can be integrated into routine primary HIV care for this underserved population.
